# A compendium of ruminant gastrointestinal phage genomes revealed a higher proportion of lytic phages than in any other environments

**DOI:** 10.1186/s40168-024-01784-2

**Published:** 2024-04-04

**Authors:** Yingjian Wu, Na Gao, Chuqing Sun, Tong Feng, Qingyou Liu, Wei-Hua Chen

**Affiliations:** 1 Key Laboratory of Molecular Biophysics of the Ministry of Education, Hubei Key Laboratory of Bioinformatics and Molecular-Imaging, Center for Artificial Biology, Department of Bioinformatics and Systems Biology, College of Life Science and Technology, Huazhong University of Science and Technology, Wuhan 430074, Hubei, China; 2 Department of Laboratory Medicine, Zhongnan Hospital of Wuhan University, Wuhan University, Wuhan, 430071 China; 3 Guangdong Provincial Key Laboratory of Animal Molecular Design and Precise Breeding, School of Life Science and Engineering, Foshan University, Foshan, 528225 China; 4 State Key Laboratory for Conservation and Utilization of Subtropical Agro-Bioresources, Guangxi University, Nanning, 530005 China; 5 Institution of Medical Artificial Intelligence, Binzhou Medical University, Yantai, 264003 China

**Keywords:** Ruminants, Gastrointestinal tract, Microbiome, Virome, Bacteriophages, Methane production

## Abstract

**Background:**

Ruminants are important livestock animals that have a unique digestive system comprising multiple stomach compartments. Despite significant progress in the study of microbiome in the gastrointestinal tract (GIT) sites of ruminants, we still lack an understanding of the viral community of ruminants. Here, we surveyed its viral ecology using 2333 samples from 10 sites along the GIT of 8 ruminant species.

**Results:**

We present the Unified Ruminant Phage Catalogue (URPC), a comprehensive survey of phages in the GITs of ruminants including 64,922 non-redundant phage genomes. We characterized the distributions of the phage genomes in different ruminants and GIT sites and found that most phages were organism-specific. We revealed that ~ 60% of the ruminant phages were lytic, which was the highest as compared with those in all other environments and certainly will facilitate their applications in microbial interventions. To further facilitate the future applications of the phages, we also constructed a comprehensive virus-bacteria/archaea interaction network and identified dozens of phages that may have lytic effects on methanogenic archaea.

**Conclusions:**

The URPC dataset represents a useful resource for future microbial interventions to improve ruminant production and ecological environmental qualities. Phages have great potential for controlling pathogenic bacterial/archaeal species and reducing methane emissions. Our findings provide insights into the virome ecology research of the ruminant GIT and offer a starting point for future research on phage therapy in ruminants.

Video Abstract

**Supplementary Information:**

The online version contains supplementary material available at 10.1186/s40168-024-01784-2.

## Background

Ruminants have been an important part of human society for centuries [1, 2], providing us with a variety of economic products including meat, milk, and fur [3]. The unique digestive system of the multi-chambered stomach including the rumen, reticulum, omasum, and abomasum allows them to return the semi-digested food fibers to the mouth for further digestion [4]. This makes them well-suited to grazing on pastures and other types of land that are not suitable for other forms of agriculture [5].

Recent research has shown that the gastrointestinal tract (GIT) of ruminants contains a great diversity of prokaryotic and eukaryotic microorganisms [6–9]. Due to the various compositions of microorganisms among different locations, the microbiomes distributed in different sites play different roles but perform equally important physiological functions in ruminants’ survival [10, 11]. The GIT microbes enable the ruminants to digest lignocellulose and other plant feedstuffs [12, 13] and protect the animal host from harmful bacteria and other pathogens [14]. However, there can also be negative effects of the GIT microbiome on ruminant health and productivity. For example: a disruption in the microbial balance in the rumen can lead to the overproduction of lactic acid, which can lower the pH and cause ruminal acidosis, a common metabolic disorder in ruminants [15]. Precision regulation of the gastrointestinal tract microbiome in ruminants is crucial for improving animal health and productivity, and reducing the environmental impact of animal agriculture. Methane-producing archaea exist in the GIT of ruminants and are one of the main sources of greenhouse gases, which have been targeted for eradication or reduction [16, 17]. However, achieving precision regulation of methane-producing archaea or pathogenic bacteria in ruminants is not a simple task, as it requires a deep understanding of the complex interplay between the microbiome, diet, and host physiology. Currently, there is a lack of system tools available to enable precise manipulation of the microbiome in ruminants [18].

Bacteriophages (phages) are a critical component of the ruminant GIT microbiome and play crucial roles in shaping microbial composition [19]. In addition, phages hold great promise for the precision manipulation of the bacteriome (i.e., the bacterial and archaeal microbes) because of their narrow microbial-host range (i.e., often at species and even strain levels [20, 21]), providing alternative ways to suppress pathogenic bacterial/archaeal species [22] and control methane emissions [16, 23]. The lifestyles of viruses can be broadly classified into two categories: lytic and lysogenic. The separation of lytic phages is important in practical applications, as lytic phages are typically more convenient to work with and have more immediate applications, such as using phages as antimicrobials against bacterial infections in animals. For example, phages have been used to control bacterial infections in dairy cattle with mastitis, which is a common and costly disease in the dairy industry [24]. Despite tremendous success in identifying viruses from various environmental sources such as the ruminants rumen [25–31], human gut [21, 32–38], aquatic, terrestrial, plants, as well as other mammals (i.e., IMG/VR v3 [39]), the virome structure remains the “dark matter” in different ruminant GIT sites, especially those other than the rumen, compared with other environments. A comprehensive resource reference phage genome is required to further characterize the viral community of the ruminant GIT and enable genome-resolution research across ruminants.

Here, we present the Unified Ruminant Phage Catalogue (URPC), a comprehensive survey of phages in the gastrointestinal tracts of ruminants. Currently, the URPC contains 64,922 non-redundant phage genomes identified using 2333 bulk metagenomics sequencing samples from 18 published works (Table S1), covering ten gastrointestinal sites from eight different ruminant species. We found that 60.53% (*n* = 39,300) of phage genomes were novel compared with those in the public viral datasets, supporting the novelty of our dataset. We characterized the distributions of the phage genomes in different ruminants and GIT sections, as well as the lifestyles of the phages. Strikingly, we revealed that ~ 60% of the ruminant phages were lytic, which was the highest as compared with those in all other environments and certainly will facilitate their applications in microbial interventions. To further facilitate the future applications of the phage, we also constructed a comprehensive virus-bacteria/archaea interaction network and identified dozens of phages that may have lytic effects on methanogenic bacteria. Together, our URPC dataset represents a useful resource for future microbial interventions to improve ruminant production and ecological environmental qualities.

## Methods

### Data collection, quality control, and removal of host- and food-associated genomes

To perform a comprehensive search for phages of the ruminant gastrointestinal tract (GIT), publicly available sequencing reads of 2333 ruminant metagenomic samples were downloaded from the National Center for Biotechnology Information/NLM/NIH (NCBI) (Figure S1; Table S1), covering eight ruminants (buffalo, camel, cattle, cow, deer, goat, sheep, yak) and ten GIT sites (rumen, reticulum, omasum, abomasum, duodenum, jejunum, ileum, cecum, colon and rectum/feces) (Table S2). Raw reads were trimmed by Trimmomatic (v 0.39) [40] with the options ‘ILLUMINACLIP: TruSeq3-PE.fa:2:30:10 SLIDINGWINDOW:4:15 MINLEN:50 LEADING:3 TRAILING:3′. To decrease potential DNA contamination from the animal hosts, reads that could be aligned to their closest genomes from NCBI (*Capra hircus*, GCF_001704415.1; *Bubalus bubalis*, GCA_004794615.1; *Camelus bactrianus*, GCF_000767855.1; Camelus dromedarius, GCF_000803125.2; *Bos taurus*,GCF_002263795.1; *Capra hircus*, GCF_001704415.1; *Alces alces*, GCA_007570765.1; *Cervus elaphus*, GCF_910594005.1; *Rangifer tarandus caribou*, GCA_019903745.1; *Capreolus capreolus*,GCA_000751575.1; *Ovis aries*, GCF_016772045.1; *Hydropotes inermis*, GCA_020226075.1; *Bos grunniens*, GCA_005887515.2), and some food-associated genomes such as *Glycine max*, *Zea Mays*, and *Medicago truncatula* were filtered out using Bowtie2 (v 2.3.5.1) [41] with options ‘–very-sensitive’. The remaining paired reads were then used for further analyses.

### Metagenomic assembly and viral contigs prediction

Unless otherwise stated, default parameters were used. Each sample was assembled using MEGAHIT (v 1.2.8) [42] with options ‘–min-contig-len 1000’. Assembled contigs of ≥ 1.5 kb in size were used to identify viral sequences using VirSorter2 (v 2.1) [43] with options ‘–include-groups “dsDNAphage, ssDNA” –min-score 0.7’ and VirFinder (v 1.1) [44] with default parameters. Contigs were identified as phages by both VirSorter2 and VirFinder (score ≥ 0.6 and *p* < 0.05).

### Quality evaluation of phage genomes and dereplication of URPC datasets

The completeness of the viral contigs was estimated using CheckV (v 0.8.1) [45]. A total of 74,519 identified viral contigs with > 50% completeness were then selected and renamed according to their animal hosts. The sequences of these contigs were merged into a single file and dereplicated using CD-HIT [46] (v4.8.1, parameters: -c 0.95 -n 8) using a global identity threshold of 95%. The resulting non-redundant representative viral genomes consisted of a total of 64,922 viral populations (VPs) and were referred to as the Unified Ruminant Phage Catalogue (URPC).

### Comparing the URPC genomes with public viral datasets

To estimate the proportion of novel phage genomes in the URPC genomes, the BLASTn tool (v 2.5.0) [47] was used to search all its sequences against a list of public viral databases including four public rumen virome datasets from the rumen virome database (RVD) [31], Hitch et al. [25] Solden et al. [26] and Friedersdorff et al. [27], NCBI viral Reference genomes, Release 201 (July 06, 2020), IMG/VR v3 [39], and four public human virome datasets such as GVD [33], GPD [32], MGV [21], and CHGV [34] (Table S3).

Average nucleotide identity (ANI) was calculated by merging the BLASTn hit regions with identity ≥ 90% and hit length ≥ 500 bp, then calculating the coverage of these regions. Based on the overall ANI, a viral sequence was considered to be novel if it has < 95% ANI as compared with other viral sequences.

### Clustering viral contigs into viral clusters (VCs)

The clustering of viral contigs into viral clusters (VCs) was performed using a strategy adopted from the GPD [32]. Briefly, the BLASTn algorithm with default parameters was used to search the nucleotide sequences of the URPC genomes and the environmental viral sequences of various habitats (e.g., Terrestrial, Freshwater, and Plants) in the IMG/VR v3 database against themselves for homologous sequences. An *E* value threshold of 1E − 10 was first used to filter the BLASTn results; the BLASTn query-hit pairs were further filtered to retain those with a coverage > 70% on the larger genomes and a coverage > 90% on the smaller genomes. Here, the coverage was calculated by merging the aligned fraction length of BLASTn high-scoring pair (HSP) sequences that shared at least 90% nucleotide similarity. Finally, a Markov clustering algorithm (MCL v14-137) [48] was used with an inflation value of 6.0, which took the filtered BLASTn results as input, carried out graph-based clustering, and clustered the viral contigs into 55,635 VCs.

### Prediction of viral lifestyles

The lifestyle classifications of all the URPC genomes were analyzed using DeePhage v1.0 [49] with the default parameters**.** DeePhage uses a scoring system to classify phage genomes into four categories, including temperate (with scores ≤ 0.3), uncertain temperate (0.3 ~ 0.5), uncertain virulent (0.5 ~ 0.7), and virulent (> 0.7). Higher scores indicate higher virulence. According to a benchmark study [50], DeePhage can classify short contigs from metagenomic data and has the best-reported performance on lifestyle prediction, while BACPHLIP [51] is only designed for complete phage genomes. And DeePhage has better generalization ability on novel phages by using a deep neural network to learn features from both DNA and protein sequences of phages, while BACPHLIP relies on a set of conserved protein domains that are associated with lysogeny. Therefore, we chose DeePhage to predict the phage lifestyles of URPC.

### Taxonomic annotation of the URPC phages

To taxonomically classify the phage contigs, VirusTaxo (https://github.com/omics-lab/VirusTaxo, downloaded on 19th April 2022) [52] was used to compare the nucleotide sequences against those in the prebuilt database of VirusTaxo and assign them to a known viral genus at an entropy index threshold of < 0.5. A Demovir script (https://github.com/feargalr/Demovir; downloaded on 6th January 2022) was then used to predict family and order ranks for the remaining genomes by searching for viral marker genes at the amino acid level.

### Co-diversification analysis of phages with their animal hosts

For all VCs that contained phages from three and more animal hosts, a phylogenetic analysis was performed. In total, 80 VCs were selected. First, Prokka v1.13 [53] (–kingdom Phages) was used to annotate the phage genomes for protein-coding genes. Pan-genome analysis was carried out for each of the VCs with Roary [54] to identify core genes and create a multiFASTA alignment of core genes using MAFFT [55] by using the multiFASTA alignment as input. A phylogenetic tree was then built using FastTree [56] v2.1.10 with default parameters. All the phylogenetic trees were then visualized and annotated using iTol [57]. For each VC, the branch length between any two phage genomes was calculated. Two single-tailed Wilcoxon rank sum tests were performed on the branch lengths from the same animal hosts and those from different animal hosts. The *p* value of the hypothesis that phages from different animal hosts had higher branch lengths was used to determine whether the phages significantly co-evolved or not co-evolved with their animal hosts.

### Microbial host analysis of the URPC phages

To find putative microbial hosts for the URPC phages, ruminant metagenome-assembled genomes (MAGs) from four publications were downloaded, including the buffalo GIT [6], ruminants GIT [7], cattle rumen [58], and goat GIT (NCBI SRA database PRJNA723432). In addition, MAGs from the Global Microbial Gene Catalog (GMGC) [59] that covered 14 different habitats were also downloaded. To establish phage-microbial host relationships between these MAGs and the URPC phages, two bioinformatic methods were used which included the CRISPR-spacer matches and nucleotide sequence similarity searches. The CRISPR spacers of the MAGs were identified using CRT (v 1.2) [60] and MinCED (v 0.4.2, https://github.com/ctSkennerton/minced). The union of the CRISPR spacers was then aligned to the viral populations using BLASTn (v 2.5.0) [47] with options of ‘-word_size 10 -dust no -max_target_seqs 10,000’. Matches with mismatch ≤ 1 and alignment length > 95% spacer length were retained. In addition, BLASTn was used to compare the viral populations with the MAGs. A putative viral-host relationship could be established if their nucleotide sequences shared > 90% identity over > 500 bps.

### Phylogenetic analysis of animal hosts

The phylogenetic analysis of the eight ruminant species was carried out using a method based on a previous study [61]. Briefly, the genomic sequences of the eight ruminant species were downloaded from the NCBI Genome database. Then, the universally conserved single-copy marker genes from each genome were identified using fetchMG [62]. The protein sequences of the markers were then aligned using MUSCLE [63] (-maxiters 100). To eliminate divergent regions from the resulting multiFASTA alignment, Gblocks [64] were used (parameters: -t = p -b3 = 8 -b4 = 2 -b5 = h). The maximum likelihood trees were built with RAxML [65] with default parameters.

### Statistical analysis

All statistical analyses were conducted using R (v4.0.4) with a two-sided Wilcoxon rank sum test unless otherwise stated.

## Results

### A unified catalog of 64,922 phage genomes from the ruminant gastrointestinal tract

**Fig. 1:**
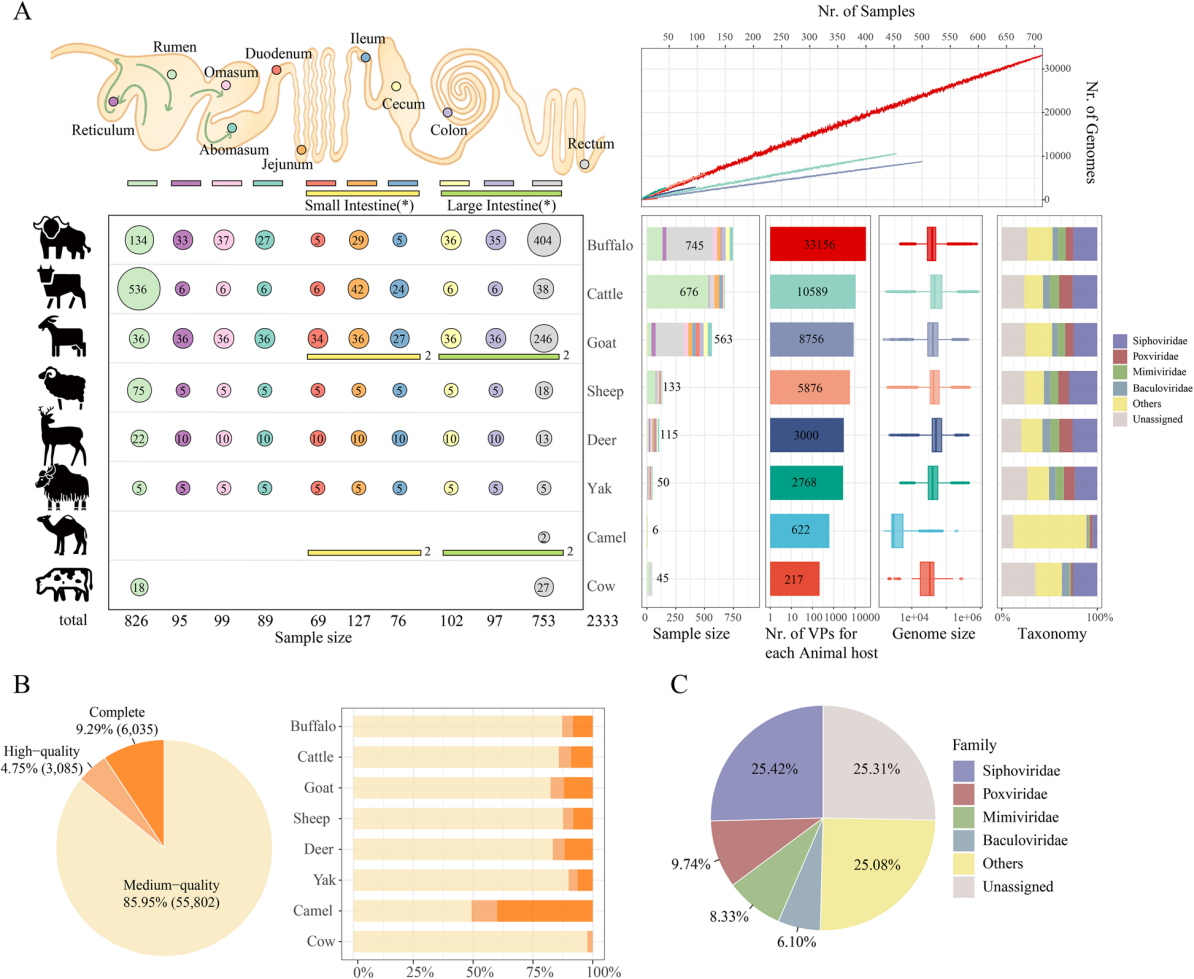
Reconstruction of the phage genomes from the ruminant gastrointestinal tract (GIT).** A** Generation of the Unified Ruminant Phage Catalogue (URPC) using 2333 GIT microbiome samples from ten GIT sites and eight ruminant species. The upper-left panel shows a graphical representation of the ruminant gastrointestinal tract (GIT), with arrows indicating the direction of food flow through the stomach. The GIT sites in this study are divided into ten sections. The bottom-left panel shows the number of samples taken from the GIT sites or sections of the ruminants. The top-right panel shows the rarefaction analysis of the unique number of VPs (*Y*-axis) as a function of collected samples (*X*-axis), while the bottom-right panel shows the statistics on the identified phages from each of the eight ruminant species, including the number, genome size and taxonomy. **B** Pie chart showing the distribution of estimated quality of the VPs in the URPC into quality tiers estimated by CheckV (complete, *n* = 6,035; high-quality, *n* = 3085; medium-quality, *n* = 55,802). Column chart showing the quality distribution of VPs in each animal host. **C** Pie chart showing the proportion of annotated VPs in the URPC at the family level by using VirusTaxo and Demovir (see “Methods)

To provide a comprehensive overview of the phages associated with the gastrointestinal tract (GIT) of ruminants, we collected a total of 2333 metagenomic samples from 18 previously published research [6, 7, 13, 23, 26, 58, 66–78] (Table S1) that covered ten GIT sites from eight ruminant species, including (Fig. 1A and Tables S1 and S2). including buffalo (*n* = 745), cattle (*n* = 930), goat (*n* = 563), sheep (*n* = 133), deer (*n* = 115), yak (*n* = 50) and cow (*n* = 46). After quality filtering and removing host DNA sequences (“Methods” section), a total of 14.17 terabytes (Tb) of clean data with more than 33 million reads and 9 billion bases per sample were retained (Table S1). We assembled them into a total of 302,721,852 contigs using MEGAHIT [42], averaging 132,251 contigs per sample with an N50 length of 3836 (Table S4).

To identify putative phage genomes, we screened the assembled contigs using a bioinformatics pipeline adopted from Luis et al. [32] (“Methods” section), followed by quality assessment for viral genome completeness using CheckV [45] and dereliction using CD-HIT [46]. We obtained a total of 74,519 viral contigs (mostly bacteriophages) with > 50% completeness and length > 1.5 kb, corresponding to 64,922 non-redundant viral populations (VPs), i.e., species-level clusters at an Average Nucleotide Identity (ANI) of 95%. We defined the latter (i.e., the 64,922 non-redundant VPs) as the Unified Ruminant Phage Catalogue (URPC). Among these, 6035 (9.29%), 3085 (4.75%), and 55,802 (85.95%) were classified as complete, high- and medium-quality, respectively, according to the CheckV tool (Fig. 1B; Table S5).

Previous studies have commonly employed a 5-kb threshold for identifying metagenome-based viral genomes [31, 39]. In our study, we opted for a 1.5-kb threshold. To substantiate this choice, we categorized all URPC phages into four length groups: < 5 kb (*n* = 777), 5 ~ 30 kb (*n* = 14,462), 30 ~ 60 kb (*n* = 35,199), and > 60 kb (*n* = 14,484). We first compared the qualities of phages in each group and were intrigued to discover that the < 5 kb group exhibited the highest proportion of complete (29.81%) phage genomes, as determined by CheckV, in comparison to the other three groups (Figure S2A). Moreover, taxonomic annotation, as per our methods, was successful for 91.48% of phages in the < 5 kb group, surpassing the rates observed in all other groups (Figure S2B). Therefore, our findings indicate that the utilization of short contigs (i.e., 1.5 ~ 5 k) not only aids in more accurately estimating the number of phages but also surprisingly enhances the annotation rates.

We used the rarefaction analysis to show that the saturation curve is far from plateaued, and more samples are required for the discovery of ruminant GIT phages (Fig. 1A). Similar trends were observed in human gut virome catalogs such as the metagenomic gut virus (MGV) [21] and a phage genome catalog of the Japanese [20]. Among all the animal hosts, we obtained the highest number of VPs (*n* = 33,156) in the buffalo, followed by the cattle (*n* = 10,589), goat (*n* = 8756), and sheep (*n* = 5876). The number of viruses identified varied in different GIT sites (Figure S3), which correlated with the number of samples we collected. The genome size and viral taxa vary among different animal hosts, indicating species-specific viral composition. Given the recent interest in human gut phageome, we then compared the genome length of URPC and other published metagenome-assembled human gut viral genomes, and found that URPC genomes were significantly longer than those in the human gut (*p* < 2.22e − 16, Wilcoxon Rank Sum test; Figure S4).

We annotated the VPs using VirusTaxo [52] and Demovir (https://github.com/feargalr/Demovir) (Fig. S5; Table S5) and assigned 74.69% of the VPs at the family level (Fig. 1C). Among the annotated VPs, 16,507 (25.42% of the total) belong to the *Siphoviridae*, followed by *Poxviridae* (*n* = 6327), *Mimiviridae* (*n* = 5409), *Baculoviridae* (*n* = 3962), *Myoviridae* (*n* = 3846), *Podoviridae* (*n* = 2360) and *Microviridae* (*n* = 2291). The overall taxonomic distribution, dominated by viral families such as *Siphoviridae*, *Microviridae*, *Myoviridae*, and *Podoviridae*, was consistent with other metagenome-derived viral catalogs in ruminant rumen (RVD) [31] and human gut [21, 33]. Particularly, we reannotated the viral genomes from RVD using our pipeline for taxonomic classification (see “Methods” section), and we found that the families *Siphoviridae*, *Podoviridae*, *Myoviridae*, *Baculoviridae*, and *Myoviridae* accounted for the majority of the viral genomes in both URPC and RVD datasets. However, we also identified more phages from the family *Podoviridae* than RVD (3.6% in URPC, and 0.5% in RVD) (Figure S6; Table S6) indicating that URPC expands the diversity of the ruminant gastrointestinal phage genomes.

We then examined the novelty of the URPC phage genomes by comparing them with several public viral databases including the NCBI viral Reference genomes (Release 201, Jul 06, 2020), IMG/VR v3 [39], four public rumen virome datasets [25–27, 31] and four human gut virome genome catalogs [21, 32–34] (Table S3). Applying an Average Nucleotide Identity (ANI) threshold of 95%, we observed that URPC exhibited the highest number of shared viral populations (VPs) with the RVD (Figure S7A). Notably, 28.11% of URPC genomes were found in the RVD, while 33.84% of RVD genomes were identified in URPC (Figure S7A). The substantial overlap between the two datasets can be attributed to the similar number of rumen samples used in URPC (826) compared to the RVD (975), despite variations in tools and criteria for viral contig identification in the latter [31] (refer to Table S7 for a detailed comparison). For a fair comparison, only 41,738 VPs from the RVD dataset meeting the same criteria as our dataset (i.e., completeness > 50%) were considered. At these criteria, this study identified a significantly higher number of VPs (64,145) compared to the RVD (Table S7).

Furthermore, with the inclusion of three additional public rumen phage datasets, a total of 46,668 (71.89%) URPC phages were determined to be novel at a 95% ANI threshold (Figure S7A), signifying URPC’s substantial contribution to expanding the ruminant gastrointestinal tract phage dataset despite prior outstanding works. When considering all the aforementioned public viral datasets, we found that 60.53% (*n* = 39,300) of VPs were considered novel at the 95% ANI threshold, indicating that the majority of URPC phages are novel (Figure S7B).

### Organism-specific distribution of URPC genomes in animal hosts

**Fig. 2:**
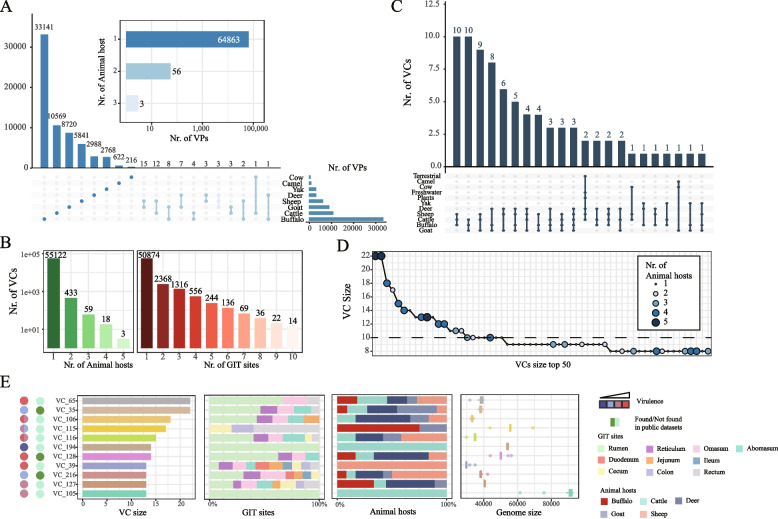
Distributions of the URPC phages in animal hosts and GIT sites.** A** The distribution of the URPC phages at the viral population (VP) level across the animal hosts. The UpSet pot shows the numbers of unique and shared VPs for the eight ruminant animals, while the bar chart shows the number of animal hosts for the VPs. **B** The distributions of the URPC phages at the viral cluster (VC) level across the animal hosts (left) and GIT sites (right). **C** An UpSet plot shows the overlaps between the broad-range phages (i.e., those that were found in two or more ruminant species) and the phage genomes collected in the IMG/VR v3 database (“Methods” section). **D** Top 50 most diverse VCs, ordered by their cluster size. The color and size of the VCs correspond to the number of animal hosts in which they were found. **E** Characterizations of the top 10 VCs, including the size, distributions in the GIT sites and animal hosts, genome size, and lifestyle. Lifestyles of phage in each VCs were predicted by DeePhage, which classified phages into four groups, including virulent (red), uncertain virulent (pink), uncertain temperate (light blue), and temperate (dark blue). Dark green and light green respectively indicate whether VCs are found in the public datasets

To investigate the correlation between the composition of VPs and their animal hosts, we first calculated the distribution of the VPs in each animal host. We discovered that 99.91% (*n* = 64,863) of VPs had only one animal host (referred to as organism-specific from now on), while only a few (*n* = 59) appeared in two or three animal hosts (Fig. 2A). To evaluate the distribution of phages with their animal hosts under higher level, we clustered the VPs into viral clusters (VCs) using methods adopted from the GPD [32] (“Methods” section) and generated a total of 55,635 VCs. Among these, 99.06% of (*n* = 55,122) the VCs are organism-specific. Similarly, most (91.43%, *n* = 50,874) of the VCs were distributed only in one GIT sit (Fig. 2B). Among the 4761 VCs that were distributed in two or more GIT sites, 92.69% (*n* = 4413) came from the same animal host, indicating an organism-specific distribution of the phages in the animal hosts. Among the 80 “broad-range” VCs (presented in three or more animal hosts), most of their animal hosts were goats (*n* = 60), buffaloes (*n* = 55), cattle (*n* = 52), sheep (*n* = 51), and deer (*n* = 39), while there were fewer in yaks (*n* = 4), camels (*n* = 2), and cows (*n* = 1) (Fig. 2C), which might be due to fewer available GIT samples of the latter three animals. To find out whether these “broad-range” VCs were food-related, we also included the VPs from the IMG/VR v3 database and re-did the viral clustering. We found that two of the “broad-range” VCs could be clustered with phages found in the terrestrial, freshwater, and plants (Fig. 2C), which confirmed previous research that phages could be readily introduced into the rumen from water sources, as well as housing and farm infrastructure [19]. However, the origins of the other 78 broad-range VCs remained to be identified.

We next characterized the largest VCs (i.e., the VCs were ranked according to the number of containing VPs). 27 out of the top 50 had two or more animal hosts, suggesting that the diverse VCs were also the well-adapted ones; this trend was more apparent among the top 10 VCs (i.e., of these, eight were found in two and more animal hosts). In addition, we observed that most of the top 10 VCs consisted of lytic bacteriophages (Fig. 2E; the lifestyles were determined using a DeePhage tool; Methods). Most of the phages in the top 10 VCs were 30 ~ 60 kb in size, which was well within the size range of typical phage genomes; in contrast, crAssphages and Gubaphages were the most diverse in the human gut virome, which was significantly longer (~ 100 kb in size) [32, 79]. Interestingly, the majority of broad-range phages in the top 10 VCs were identified in the rumen (63%; Table S5), much higher than those identified in the rectum (including fecal samples; 17%), despite that, we had comparable samples from the two GIT sites (826 vs. 753, Fig. 1A). These results strongly suggested that at least some of the rumen phages were likely originated from the environment.

In summary, we found that most of the ruminant phages are organism- and GIT site-specific at both the VP and VC levels, with a few broad-range ones, likely originating from the environment.

### Co-diversification of phages with their animal hosts

**Fig. 3:**
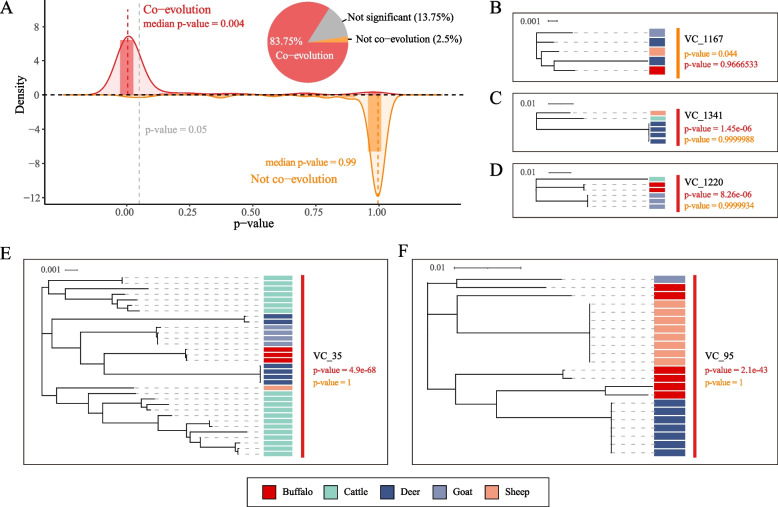
Co-evolution analysis of the broad-range VCs with the animal hosts.** A** Overall statistics of the co-evolution analysis. Here the density and bar plots show the likelihood (*p* values) distributions of the phages in the 80 broad-range VCs were co-evolved (red line and bars) or not co-evolved (orange line and bars). One-tailed Wilcoxon rank sum tests were performed on the branch lengths from the same animal hosts and different animal hosts (“Methods” section). The pie chart shows the proportion of coevolved and non-coevolved viral clusters (VCs) with three or more animal hosts. **B**–**F** Example phylogenetic trees of VCs with their animal hosts in which the phages showed significant not co-evolution (**B**) or co-evolution (**C**–**F**)

We next investigated whether the phage genomes in the broad-range VCs could show co-diversification patterns with their animal hosts. We focused on the 80 broad-range VCs that were presented in three or more animal hosts; for each of the VCs, we used a phylogenetic tree-based method to test whether phages from the same animal host were significantly closer than those of different animal hosts (“Methods” section). We observed that in 83.75% (*n* = 67) of the VCs, the phages tend to significantly co-evolute with their animal hosts (i.e., VPs from the same animal host in a VC were clustered together on the evolutionary tree and had significantly closer evolutionary distance), while only 2.5% were classified as significantly not co-evolution (Fig. 3A). We included in Fig. 3B–F afew typical examples to showcase our analysis. As shown in Fig. 3B, we identified two deer phages in VC_1167, which showed closer phylogenetic relationships to phages of other ruminants than to each other, indicating significant non-co-evolution. Conversely, Fig. 3C–F showed a few cases of significant co-evolution in VC_1341, VC_1220, VC_35, and VC_95, in which multiple phages from the same animal hosts often cluster together in their respective phylogenetic trees. Further efforts would be required to illustrate whether the co-evolution was due to the adaption of the microbial hosts of the phages to the ruminant species.

### The URPC contains the highest proportion of lytic phages as compared with other environments

**Fig. 4:**
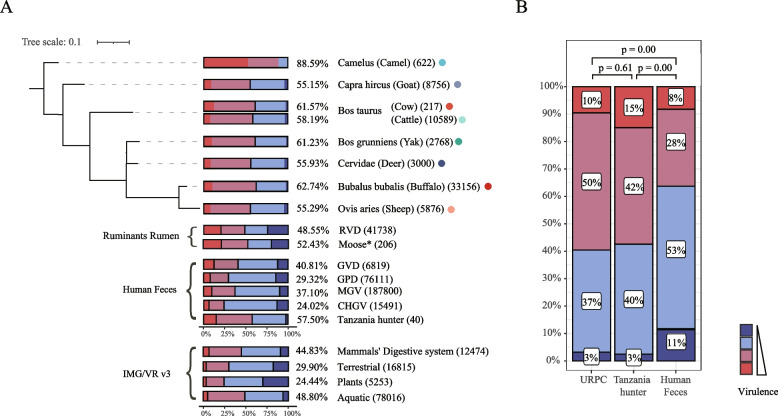
Lifestyle analysis of the phages identified in the ruminant GIT and other environments. **A** Phage lifestyle analysis of the ruminant GIT, rumen, human gut, and other habitats in the IMG/VR v3 database. Due to the previous utilization of Virsorter1 [80] for viral identification, we reannotated the viral genomes from the moose rumen [26] using our viral identification pipeline (i.e., VirSorter2, VirFinder and CheckV; see “Methods” section), which has been marked with *. A DeePhage tool was used to analyze the phage lifestyles, which classified phages into four groups, including virulent (red), uncertain virulent (pink), uncertain temperate (light blue), and temperate (dark blue). The proportions next to the bar plots indicate the overall proportion of lytic phages (i.e., the virulent and uncertain virulent combined) in each dataset, while the numbers in the parentheses indicate the overall phage numbers in the corresponding datasets that passed our filtering criteria (i.e., CheckV completeness score > 50% and length > 1.5 k). **B** Comparisons in the phage lifestyles among the ruminants, Tanzania hunters, and the combination of public human gut virome datasets including the GPD, GVD, MGV, and CHGV. *P* values were calculated using the chi-square test

The observation that the lytic phages account for seven of the top 10 VCs encouraged us to further characterize the lifestyle of the ruminant phages. Because the metagenome samples we collected were not separated viral particles, we expected that many phages were derived from bacterial cells and were more likely to be integrated into the bacterial chromosomes as temperate phages. However, we found that the majority (59.60%, *n* = 38,696) of the phages were classified as lytic phages (virulent or uncertain virulent) using a DeePhage tool [49], which outperformed several existing tools in terms of accuracy. We found similar proportions of lytic phages in the eight ruminants (55.15 to 88.59%, with an interquartile range (IQR) of 55.77 to 61.86%; Fig. 4A) as well as across GIT sites (53.76% to 61.51%, with an IQR of 57.35 to 59.08%; Figure S8). Similarly, we also identified a comparable percentage of lytic phages in two rumen viral genome datasets (48.55% in RVD, and 52.43% in moose rumen [26]) that contained at least 200 phage genomes (Table S3), further supporting our findings.

We then compared with public datasets and found surprisingly that phages from all other environments had lower proportions of lytic phages. Since the length and completeness of the virus affect the number of genes detected in the viral genome, we performed the same quality control consistent with our URPC (filtering length > 1.5 k and completeness > 50% estimated using CheckV [45]) on the viral genomes from public databases (Table S3). For example, 44.40% of phages in the IMG/VR v3, the most comprehensive phage database so far, are lytic, which is significantly lower than the URPC. We found the same results when stratifying the IMG/VR phages according to the habits (Fig. 4A). In addition, we found an overall of 32% lytic phages in several human gut virome datasets (24.02% to 40.81%, interquartile range (IQR): 28.00 to 38.03%; Table S3), consistent with previous observations that the human gut phages were mostly temperate [20, 32].

Interestingly, out of all the human gut virome/metagenome datasets we have analyzed, we found only one that contained a similar proportion of lytic phages to the URPC: the Tanzania hunter gut metagenome. As shown in Fig. 4B, 57.5% of the phages identified in the Tanzania hunter dataset were lytic, similar to that of the URPC (*p* = 0.61, chi-square test), while both were significantly different from the other human gut virome datasets (*p* < 0.01, chi-square test). We speculate that the different lifestyles between the Tanzania hunter and the other human samples might underlie the different phage lifestyles, such as the consumption of raw or less processed foods and high exposure to microbe-enriched environments of the hunters [81]. However, due to the limited numbers of samples (i.e., 40 metagenomic samples from the NCBI SRA database; PRJNA392180) and identified viral contigs (i.e., 40 non-redundant viral contigs with completeness above 50%), our hypothesis should be tested using larger datasets.

### Bacterial and archaeal host prediction of the URPC phages identifies dozens of lytic phages targeting methane-producers

**Fig. 5:**
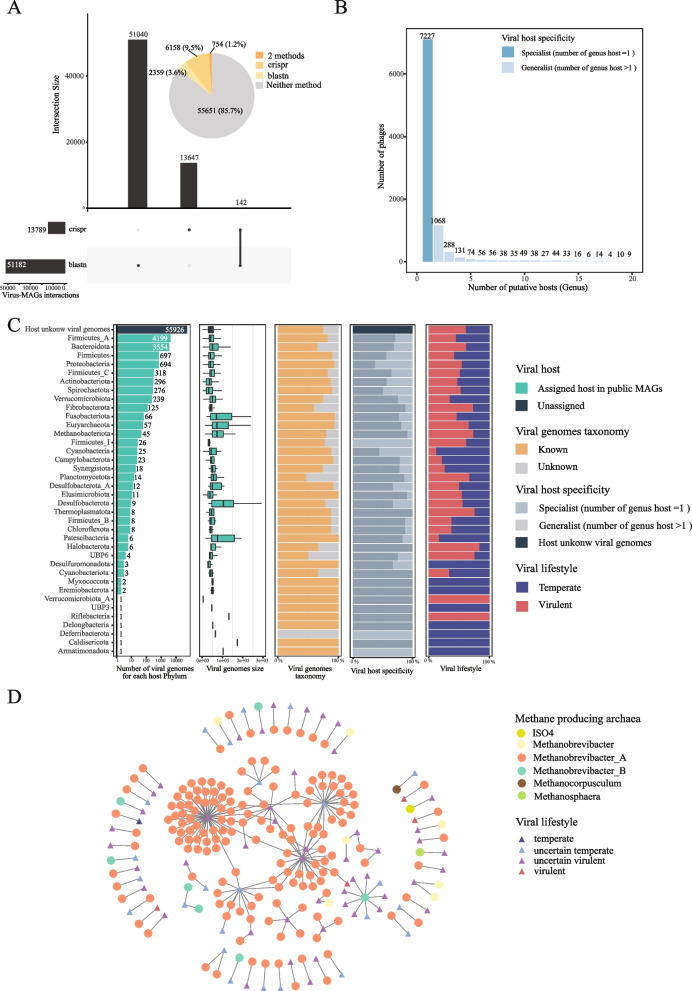
Host prediction of the ruminant GIT phages and identification of lytic phages targeting methane producers.** A** Statistics on the viral-microbial host relationships using two different methods, namely CRISPR-spacer and sequence homology-(blastn) based methods. The UpSet plot shows the number of unique and shared viral-host interactions according to the two methods. The pie chart shows the proportion of phages whose host(s) could be predicted by these methods. **B** Histogram showing the number of phages (*Y*-axis) as a function of the number of predicted hosts at the genus level (*X*-axis). The phages could be divided into a specialist (number of host genus = 1) and a generalist (number of host genera > 1). **C** Characteristics of the phages stratified by their predicted microbial hosts at the phylum level, including the genome size, annotation rate, host specificity, and lifestyles. The lifestyles were predicted using DeePhage and classified into two groups (virulent: DeePhage score >  = 0.5, temperate: score < 0.5).** D** The interaction network between phages and methane-producers (i.e., archaea) predicted by phage-host relationships

Predicting viral hosts is crucial for understanding their roles and impacts [82], and phages can serve as ideal tools to regulate ruminant GIT ecosystems by limiting the number of their microbial hosts through lytic infections [83]. We thus predicted hosts for all the VPs using metagenome-assembled genomes (MAGs) from public datasets [6, 7, 58, 59] using two different methods, namely the CRISPR-spacer- and sequence similarity-based methods (“Methods” section). We were able to assign a total of 9271 phages (14.28% out of the total) to their putative bacterial/archaeal hosts, including 4690 (50.59%) to a public ruminant GIT genome collection and 5562 (59.99%) to the MAGs in the Global Microbial Gene Catalog (GMGC [59]). Among these, 754 phages could be assigned consistently to the same hosts by both methods (e.g., the highly confident prediction results; Fig. 5A, Table S8). We observed little overlaps between the predicted virus-host connection pairs produced by the two methods, consistent with previous results [84, 85]. In general, a total of 7227 (77.95%) phages were classified as specialist (Fig. 5B), meaning that they infect only one genus (i.e., specialist phages), while the others were predicted to infect two or more genera (i.e., generalist phages), which confirmed previous research that phages have a limited host range [20, 86, 87].

Among all the predicted virus-microbial host relationships, *Firmicutes* (the combination of *Firmicutes_A*, *Firmicutes,* and *Firmicutes_C*) was the most common phylum targeted by the URPC phages (*n* = 5,214, i.e., the number of interacting phages), followed by *Bacteroidetes* (*n* = 3554) (Fig. 5C), which were the two groups of beneficial bacteria that were dominant in the ruminant GIT [88]. Many of the functionally important genera were targeted by the phages. At the genus level, the most predicted hosts were *Prevotella* (*n* = 1035; one of the most abundant and versatile genera that contribute to hemicellulose degradation, lignocellulose pretreatment, and feruloyl esterase activity [89]), followed by cellulose digestive *Bacteroides* (*n* = 625) which secrete cellulases and hemicellulases to degrade cellulose and hemicellulose into glucose and other sugars for ruminants [12, 90], *Lachnospira* (*n* = 524) and *Roseburia* (*n* = 381) major short-chain fatty acids (SCFAs) producers in the rumen providing energy and anti-inflammatory effects [12, 91, 92]. Our results suggested important regulatory roles of phages in the ruminant GIT microbial structures and functions.

Phage could be an ideal tool to inhibit the growth of methane-producing archaea in the GIT of ruminants [93]. However, no lytic phages targeting methane producers have been identified [16]. Here, we retrieved 109 phages that infected methanogenic archaea from the phage-microbial host analysis (Fig. 5D). Of these, 74 were lytic (virulent or uncertain-virulent; “Method” section) and could target the six genera of methanogens (i.e., *ISO4*, *Methanobrevibacter*, *Methanobrevibacter_A*,* Methanobrevibacter_B*,* Methanocorpusculum*, *Methanosphaera*) annotated by GTDB-Tk [94]. These results should facilitate targeted isolation of phages and experimental validation of their lysis efficiency against methanogenic archaea.

## Discussion

Many ruminant animals are important livestock and have more complicated gastrointestinal tracts (GITs) than other mammals. It has been well established that the GIT microbiome plays important roles in not only feedstuff digestion and absorption [12, 14], but also the development, health, and diseases as well as the quality of animal products such as meat, milk, and fur [3]. So far, there has been significant progress in the study of ruminant microbiomes, particularly bacteria/archaea [6–8]. However, we still lack systematic tools to precisely manipulate the microbiomes to improve the wellness of the animals and the qualities of their products. Phages (bacteriophages and archaeal viruses), especially lytic ones, are ideal tools for such purposes because of their abundance in nature and high microbial host specificity [93]. However, there is still a lack of comprehensive research on ruminant phages, especially at GIT sites other than the rumen [25–31]. In this study, we filled this gap by mining 2333 metagenome samples from eight ruminant species, covering all major sites along the GIT (ten sites, including the rumen). Based on the data, we constructed a Unified Ruminant Phage Catalogue (URPC) comprising 64,922 phage genomes. Of which, 60.53% were novel as compared with public virome databases, indicating that the URPC represents a significant expansion to ruminant GIT phages and is the most comprehensive dataset so far.

We first examined the distributions of the URPC in the eight ruminants and across different GIT sites. Broad-range phages, i.e., those found in multiple hosts are of higher values because they could be applied to multiple animals, e.g., to kill pathogens. However, we found that most phages were organism-specific, which was expected given the results that the rarefaction curve was far from saturation (Fig. 1A) and consistent with the previous observations in humans that the gut virome was often individual-specific [20, 32]. Nevertheless, these results also indicate that we have a much larger pool of arsenals from which we can find phages targeting specific bacterial/archaeal species of interest.

Lytic phages often have higher application potentials because they are easier to isolate and more efficient in killing their microbial hosts. Surprisingly, we found that ~ 60% of the URPC phages are lytic, higher than any other environments we have surveyed, including the terrestrial, marine, aquatic, freshwater, plants, and human gut (Fig. 4A). Moreover, we also observed a similar elevated proportion of lytic phages within two rumen viral genome dataset (RVD and moose rumen [26]). Lytic phages are often isolated from the sewage [22]; our results thus provided better alternatives for lytic phage isolation.

To further facilitate future application of the URPC phages, we predicted their microbial hosts using public MAG datasets, including several ruminant GIT MAGs and those of the other environments. Of particular interest, we obtained 109 phages targeting methane-producing species in all six archaeal genera by mining the phage-host relationships; of which 74 were lytic ones. Previous studies have shown that phages targeting methanogens may help reduce methane emissions [13], but we lack a large-scale method for identifying such phages [95]. Therefore, our results will facilitate the targeted isolation of lytic phages against methanogens and other bacterial/archaeal species in general.

Overall, our assembly and analysis of the URPC phages massively expanded the ruminant GIT phages and paved the way for microbiome intervention to improve the ruminant and environmental quality.

## Conclusions

We filled the gap in ruminant viral ecology research by providing a catalog of phage genomes and identifying many lytic viruses that could target methane producers. Our findings provide insights into the phage community of the ruminant GIT and can be used as a starting point for future research on microbiome manipulation in ruminants.

### Supplementary Information

**Additional file 1:** **Table S1.** Detailed sampling information for 2333 samples. **Table S2.** Number of ruminant gastrointestinal tract samples. **Table S3.** Detailed information for public viral reference databases used in this study. **Table S4.** Statistics of assembly results of 2333 samples. **Table S5.** Statistical information and classification level annotation of 64,922 phages. **Table S6.** Comparison of methods between URPC and RVD. **Table S7.** Classification level annotation of 41,738 viral genomes of RVD. **Table S8.** An overview of URPC phages and their bacterial/archaeal host prediction.

**Additional file 2:** **Figure S1.** Overview of the custom pipeline employed for the reconstruction of URPC. **Figure S2.** Phage quality assessment and annotation at different lengths. Figure S3. The number of phages identified along the ruminant gastrointestinal tract.  **Figure S4.** The URPC shows longer length than viral genomes from human gut. **Figure S5.** Taxonomic annotation of the URPC genomes. **Figure S6.** Family annotation of RVD viral genomes by VirusTaxo and Demovir. **Figure S7.** Percentages of novel phages in URPC as compared with public datasets at 95% average nucleotide identity (ANI) thresholds. **Figure S8.** Lifestyle analysis of the phages identified in the ruminants of different GIT sites.

## Data Availability

The Unified Ruminant Phage Catalogue (URPC) used in this study is available in the Figshare database under accession code 22,219,093 (https://doi.org/10.6084/m9.figshare.22219093.v2).

## References

[1] Oltjen JW, Beckett JL (1996). Role of ruminant livestock in sustainable agricultural systems. J Anim Sci.

[2] Chen L, Qiu Q, Jiang Y, Wang K, Lin Z, Li Z, Bibi F, Yang Y, Wang J, Nie W, et al*.* Large-scale ruminant genome sequencing provides insights into their evolution and distinct traits. Science. 2019;364(6446):eaav6202. 10.1126/science.aav6202 31221828

[3] Matthews C, Crispie F, Lewis E, Reid M, O'Toole PW, Cotter PD (2019). The rumen microbiome: a crucial consideration when optimising milk and meat production and nitrogen utilisation efficiency. Gut Microbes.

[4] Liu K, Zhang Y, Yu Z, Xu Q, Zheng N, Zhao S, Huang G, Wang J (2021). Ruminal microbiota-host interaction and its effect on nutrient metabolism. Anim Nutr.

[5] Eisler MC, Lee MR, Tarlton JF, Martin GB, Beddington J, Dungait JA, Greathead H, Liu J, Mathew S, Miller H (2014). Agriculture: Steps to sustainable livestock. Nature.

[6] Tong F, Wang T, Gao NL, Liu Z, Cui K, Duan Y, Wu S, Luo Y, Li Z, Yang C (2022). The microbiome of the buffalo digestive tract. Nat Commun.

[7] Xie F, Jin W, Si H, Yuan Y, Tao Y, Liu J, Wang X, Yang C, Li Q, Yan X (2021). An integrated gene catalog and over 10,000 metagenome-assembled genomes from the gastrointestinal microbiome of ruminants. Microbiome.

[8] Perez-Barberia FJ, Elston DA, Gordon IJ, Illius AW (2004). The evolution of phylogenetic differences in the efficiency of digestion in ruminants. Proc Biol Sci.

[9] Sun Y, Sun P, Hu Y, Shan L, Geng Q, Gong Y, Fan H, Zhang T, Zhou Y (2022). Elevated testicular apoptosis is associated with elevated sphingosine driven by gut microbiota in prediabetic sheep. BMC Biol.

[10] Zhang Y, Choi SH, Nogoy KM, Liang S (2021). Review: The development of the gastrointestinal tract microbiota and intervention in neonatal ruminants. Animal.

[11] Huws SA, Edwards JE, Lin W, Rubino F, Alston M, Swarbreck D, Caim S, Stevens PR, Pachebat J, Won MY (2021). Microbiomes attached to fresh perennial ryegrass are temporally resilient and adapt to changing ecological niches. Microbiome.

[12] Xu Q, Qiao Q, Gao Y, Hou J, Hu M, Du Y, Zhao K, Li X (2021). Gut Microbiota and Their Role in Health and Metabolic Disease of Dairy Cow. Front Nutr.

[13] Cao Y, Feng T, Wu Y, Xu Y, Du L, Wang T, Luo Y, Wang Y, Li Z, Xuan Z (2023). The multi-kingdom microbiome of the goat gastrointestinal tract. Microbiome.

[14] Fu Y, He Y, Xiang K, Zhao C, He Z, Qiu M, Hu X, Zhang N. The Role of Rumen Microbiota and Its Metabolites in Subacute Ruminal Acidosis (SARA)-Induced Inflammatory Diseases of Ruminants. Microorganisms. 2022;10(8):1495. 10.3390/microorganisms10081495 PMC9332062 35893553

[15] Elmhadi ME, Ali DK, Khogali MK, Wang H (2022). Subacute ruminal acidosis in dairy herds: Microbiological and nutritional causes, consequences, and prevention strategies. Anim Nutr.

[16] Mizrahi I, Wallace RJ, Morais S (2021). The rumen microbiome: balancing food security and environmental impacts. Nat Rev Microbiol.

[17] Lovendahl P, Difford GF, Li B, Chagunda MGG, Huhtanen P, Lidauer MH, Lassen J, Lund P (2018). Review: Selecting for improved feed efficiency and reduced methane emissions in dairy cattle. Animal.

[18] Huws SA, Creevey CJ, Oyama LB, Mizrahi I, Denman SE, Popova M, Munoz-Tamayo R, Forano E, Waters SM, Hess M (2018). Addressing Global Ruminant Agricultural Challenges Through Understanding the Rumen Microbiome: Past, Present, and Future. Front Microbiol.

[19] Gilbert RA, Townsend EM, Crew KS, Hitch TCA, Friedersdorff JCA, Creevey CJ, Pope PB, Ouwerkerk D, Jameson E (2020). **Ru**men virus populations: technological advances enhancing current understanding. Front Microbiol.

[20] Nishijima S, Nagata N, Kiguchi Y, Kojima Y, Miyoshi-Akiyama T, Kimura M, Ohsugi M, Ueki K, Oka S, Mizokami M (2022). Extensive gut virome variation and its associations with host and environmental factors in a population-level cohort. Nat Commun.

[21] Nayfach S, Paez-Espino D, Call L, Low SJ, Sberro H, Ivanova NN, Proal AD, Fischbach MA, Bhatt AS, Hugenholtz P, Kyrpides NC (2021). Metagenomic compendium of 189,680 DNA viruses from the human gut microbiome. Nat Microbiol.

[22] Jin M, Chen J, Zhao X, Hu G, Wang H, Liu Z, Chen WH (2022). **A**n engineered lambda phage enables enhanced and strain-specific killing of enterohemorrhagic Escherichia coli. Microbiol Spectr.

[23] Shi W, Moon CD, Leahy SC, Kang D, Froula J, Kittelmann S, Fan C, Deutsch S, Gagic D, Seedorf H (2014). Methane yield phenotypes linked to differential gene expression in the sheep rumen microbiome. Genome Res.

[24] Sharun K, Dhama K, Tiwari R, Gugjoo MB, Iqbal Yatoo M, Patel SK, Pathak M, Karthik K, Khurana SK, Singh R (2021). Advances in therapeutic and managemental approaches of bovine mastitis: a comprehensive review. Vet Q.

[25] Hitch TCA, Edwards JE, Gilbert RA. Metatranscriptomics reveals mycoviral populations in the ovine rumen. FEMS Microbiol Lett. 2019;366(13):fnz161. 10.1093/femsle/fnz161 31314094

[26] Solden LM, Naas AE, Roux S, Daly RA, Collins WB, Nicora CD, Purvine SO, Hoyt DW, Schuckel J, Jorgensen B (2018). Interspecies cross-feeding orchestrates carbon degradation in the rumen ecosystem. Nat Microbiol.

[27] Friedersdorff JCA, Kingston-Smith AH, Pachebat JA, Cookson AR, Rooke D, Creevey CJ (2020). The Isolation and Genome Sequencing of Five Novel Bacteriophages From the Rumen Active Against Butyrivibrio fibrisolvens. Front Microbiol.

[28] Magossi G, Holman DB, Schmidt KN, Hoselton SA, Amat S (2023). Genome sequences of 11 Alkalihalobacillus clausii, Bacillus safensis, and Escherichia coli bacteriophages isolated from bovine rumen and vagina. Microbiol Resour Announc.

[29] Namonyo S, Wagacha M, Maina S, Wambua L, Agaba M (2018). A metagenomic study of the rumen virome in domestic caprids. Arch Virol.

[30] Anderson CL, Sullivan MB, Fernando SC (2017). Dietary energy drives the dynamic response of bovine rumen viral communities. Microbiome.

[31] Yan M, Pratama AA, Somasundaram S, Li Z, Jiang Y, Sullivan MB, Yu Z (2023). Interrogating the viral dark matter of the rumen ecosystem with a global virome database. Nat Commun.

[32] Camarillo-Guerrero LF, Almeida A, Rangel-Pineros G, Finn RD, Lawley TD: Massive expansion of human gut bacteriophage diversity. Cell.2021;184(4):1098–1109 e1099. 10.1016/j.cell.2021.01.029 PMC7895897 33606979

[33] Gregory AC, Zablocki O, Zayed AA, Howell A, Bolduc B, Sullivan MB: The gut virome database reveals age-dependent patterns of virome diversity in the human gut. Cell Host Microbe 2020;28(5):724–740 e728. 10.1016/j.chom.2020.08.003 PMC7443397 32841606

[34] Chen W-H, Chen J, Sun C, Dong Y, Jin M, Lai S, Jia L, Zhao X, Gao NL, Liu Z *et al*: Efficient recovery of complete gut phage genomes by combined short- and long-sequencing. bioRxiv 2022:2022.2007.2003.498593. 10.1002/advs.202305818 PMC10987132 38240578

[35] Tisza MJ, Buck CB. A catalog of tens of thousands of viruses from human metagenomes reveals hidden associations with chronic diseases. Proc Natl Acad Sci USA. 2021;118(23):e2023202118. 10.1073/pnas.2023202118 PMC8201803 34083435

[36] Lai S, Jia L, Subramanian B, Pan S, Zhang J, Dong Y, Chen WH, Zhao XM (2021). mMGE: a database for human metagenomic extrachromosomal mobile genetic elements. Nucleic Acids Res.

[37] Van Espen L, Bak EG, Beller L, Close L, Deboutte W, Juel HB, Nielsen T, Sinar D, De Coninck L, Frithioff-Bojsoe C *et al*: A previously undescribed highly prevalent phage identified in a danish enteric virome catalog. mSystems 2021;6(5):e0038221. 10.1128/mSystems.00382-21 PMC8525569 34665009

[38] Li J, Yang F, Xiao M, Li A (2022). Advances and challenges in cataloging the human gut virome. Cell Host Microbe.

[39] Roux S, Paez-Espino D, Chen IA, Palaniappan K, Ratner A, Chu K, Reddy TBK, Nayfach S, Schulz F, Call L (2021). IMG/VR v3: an integrated ecological and evolutionary framework for interrogating genomes of uncultivated viruses. Nucleic Acids Res.

[40] Bolger AM, Lohse M, Usadel B (2014). Trimmomatic: a flexible trimmer for Illumina sequence data. Bioinformatics.

[41] Langmead B, Salzberg SL (2012). Fast gapped-read alignment with Bowtie 2. Nat Methods.

[42] Li D, Liu CM, Luo R, Sadakane K, Lam TW (2015). MEGAHIT: an ultra-fast single-node solution for large and complex metagenomics assembly via succinct de Bruijn graph. Bioinformatics.

[43] Guo J, Bolduc B, Zayed AA, Varsani A, Dominguez-Huerta G, Delmont TO, Pratama AA, Gazitua MC, Vik D, Sullivan MB, Roux S (2021). VirSorter2: a multi-classifier, expert-guided approach to detect diverse DNA and RNA viruses. Microbiome.

[44] Ren J, Ahlgren NA, Lu YY, Fuhrman JA, Sun FZ (2017). VirFinder: a novel k-mer based tool for identifying viral sequences from assembled metagenomic data. Microbiome.

[45] Nayfach S, Camargo AP, Schulz F, Eloe-Fadrosh E, Roux S, Kyrpides NC (2021). CheckV assesses the quality and completeness of metagenome-assembled viral genomes. Nat Biotechnol.

[46] Fu L, Niu B, Zhu Z, Wu S, Li W (2012). CD-HIT: accelerated for clustering the next-generation sequencing data. Bioinformatics.

[47] Camacho C, Coulouris G, Avagyan V, Ma N, Papadopoulos J, Bealer K, Madden TL (2009). BLAST+: architecture and applications. BMC Bioinformatics.

[48] Enright AJ, Van Dongen S, Ouzounis CA (2002). An efficient algorithm for large-scale detection of protein families. Nucleic Acids Res.

[49] Wu S, Fang Z, Tan J, Li M, Wang C, Guo Q, Xu C, Jiang X, Zhu H. DeePhage: distinguishing virulent and temperate phage-derived sequences in metavirome data with a deep learning approach. Gigascience. 2021;10(9):giab056. 10.1093/gigascience/giab056 PMC8427542 34498685

[50] Shang J, Tang X, Sun Y. PhaTYP: predicting the lifestyle for bacteriophages using BERT. Brief Bioinform. 2023;24(1):bbac487. 10.1093/bib/bbac487 PMC9851330 36659812

[51] Hockenberry AJ, Wilke CO (2021). BACPHLIP: predicting bacteriophage lifestyle from conserved protein domains. PeerJ.

[52] Raju RS, Al Nahid A, Chondrow Dev P, Islam R (2022). VirusTaxo: Taxonomic classification of viruses from the genome sequence using k-mer enrichment. Genomics.

[53] Seemann T (2014). Prokka: rapid prokaryotic genome annotation. Bioinformatics.

[54] Page AJ, Cummins CA, Hunt M, Wong VK, Reuter S, Holden MT, Fookes M, Falush D, Keane JA, Parkhill J (2015). Roary: rapid large-scale prokaryote pan genome analysis. Bioinformatics.

[55] Katoh K, Misawa K, Kuma K, Miyata T (2002). MAFFT: a novel method for rapid multiple sequence alignment based on fast Fourier transform. Nucleic Acids Res.

[56] Price MN, Dehal PS, Arkin AP (2010). FastTree 2–approximately maximum-likelihood trees for large alignments. PLoS ONE.

[57] Letunic I, Bork P (2021). Interactive Tree Of Life (iTOL) v5: an online tool for phylogenetic tree display and annotation. Nucleic Acids Res.

[58] Stewart RD, Auffret MD, Warr A, Walker AW, Roehe R, Watson M (2019). Compendium of 4,941 rumen metagenome-assembled genomes for rumen microbiome biology and enzyme discovery. Nat Biotechnol.

[59] Coelho LP, Alves R, Del Rio AR, Myers PN, Cantalapiedra CP, Giner-Lamia J, Schmidt TS, Mende DR, Orakov A, Letunic I (2022). Towards the biogeography of prokaryotic genes. Nature.

[60] Bland C, Ramsey TL, Sabree F, Lowe M, Brown K, Kyrpides NC, Hugenholtz P (2007). CRISPR recognition tool (CRT): a tool for automatic detection of clustered regularly interspaced palindromic repeats. BMC Bioinformatics.

[61] Chen WH, Lu G, Bork P, Hu S, Lercher MJ (2016). Energy efficiency trade-offs drive nucleotide usage in transcribed regions. Nat Commun.

[62] Milanese A, Mende DR, Paoli L, Salazar G, Ruscheweyh HJ, Cuenca M, Hingamp P, Alves R, Costea PI, Coelho LP (2019). Microbial abundance, activity and population genomic profiling with mOTUs2. Nat Commun.

[63] Edgar RC (2004). MUSCLE: a multiple sequence alignment method with reduced time and space complexity. BMC Bioinformatics.

[64] Talavera G, Castresana J (2007). Improvement of phylogenies after removing divergent and ambiguously aligned blocks from protein sequence alignments. Syst Biol.

[65] Stamatakis A (2014). RAxML version 8: a tool for phylogenetic analysis and post-analysis of large phylogenies. Bioinformatics.

[66] Hess M, Sczyrba A, Egan R, Kim TW, Chokhawala H, Schroth G, Luo S, Clark DS, Chen F, Zhang T (2011). Metagenomic discovery of biomass-degrading genes and genomes from cow rumen. Science.

[67] Wallace RJ, Rooke JA, McKain N, Duthie CA, Hyslop JJ, Ross DW, Waterhouse A, Watson M, Roehe R (2015). The rumen microbial metagenome associated with high methane production in cattle. BMC Genomics.

[68] Rubino F, Carberry C (2017). S MW, Kenny D, McCabe MS, Creevey CJ: **Divergent functional isoforms drive niche specialisation for nutrient acquisition and use in rumen microbiome**. ISME J.

[69] Svartstrom O, Alneberg J, Terrapon N, Lombard V, de Bruijn I, Malmsten J, Dalin AM, El Muller E, Shah P, Wilmes P (2017). Ninety-nine de novo assembled genomes from the moose (Alces alces) rumen microbiome provide new insights into microbial plant biomass degradation. ISME J.

[70] Tanca A, Fraumene C, Manghina V, Palomba A, Abbondio M, Deligios M, Pagnozzi D, Addis MF, Uzzau S (2017). Diversity and functions of the sheep faecal microbiota: a multi-omic characterization. Microb Biotechnol.

[71] Wolff SM, Ellison MJ, Hao Y, Cockrum RR, Austin KJ, Baraboo M, Burch K, Lee HJ, Maurer T, Patil R (2017). Diet shifts provoke complex and variable changes in the metabolic networks of the ruminal microbiome. Microbiome.

[72] Weinroth MD, Scott HM, Norby B, Loneragan GH, Noyes NR, Rovira P, Doster E, Yang X, Woerner DR, Morley PS, Belk KE. Effects of Ceftiofur and Chlortetracycline on the Resistomes of Feedlot Cattle. Appl Environ Microbiol. 2018;84(13):e00610–18. 10.1128/AEM.00610-18 PMC6007121 29728379

[73] Al-Masaudi S, El Kaoutari A, Drula E, Redwan EM, Lombard V, Henrissat B (2019). A metagenomics investigation of carbohydrate-active enzymes along the goat and camel intestinal tract. Int Microbiol.

[74] Li F, Hitch TCA, Chen Y, Creevey CJ, Guan LL (2019). Comparative metagenomic and metatranscriptomic analyses reveal the breed effect on the rumen microbiome and its associations with feed efficiency in beef cattle. Microbiome.

[75] Lin L, Xie F, Sun D, Liu J, Zhu W, Mao S (2019). Ruminal microbiome-host crosstalk stimulates the development of the ruminal epithelium in a lamb model. Microbiome.

[76] Malmuthuge N, Liang G, Guan LL (2019). Regulation of rumen development in neonatal ruminants through microbial metagenomes and host transcriptomes. Genome Biol.

[77] Serajus S, Seon Woo K, Jeffrey SK, Ernest H, Bradd JH, Jo Ann S (2019). Metagenomic analysis of the fecal microbiomes from Escherichia coli O157:H7-shedding and non-shedding cows on a single dairy farm. Food Control.

[78] Li J, Zhong H, Ramayo-Caldas Y, Terrapon N, Lombard V, Potocki-Veronese G, Estelle J, Popova M, Yang Z, Zhang H, *et al.* A catalog of microbial genes from the bovine rumen unveils a specialized and diverse biomass-degrading environment. Gigascience. 2020;9(6):giaa057. 10.1093/gigascience/giaa057 PMC7260996 32473013

[79] Guerin E, Shkoporov A, Stockdale SR, Clooney AG, Ryan FJ, Sutton TDS, Draper LA, Gonzalez-Tortuero E, Ross RP, Hill C: Biology and Taxonomy of crAss-like Bacteriophages, the Most Abundant Virus in the Human Gut. Cell Host Microb*e* 2018;24(5):653–664 e656. 10.1016/j.chom.2018.10.002 30449316

[80] Roux S, Enault F, Hurwitz BL, Sullivan MB (2015). VirSorter: mining viral signal from microbial genomic data. PeerJ.

[81] Mallott EK, Amato KR. Butyrate production pathway abundances are similar in human and nonhuman primate gut microbiomes. Mol Biol Evol. 2022;39(1):msab279. 10.1093/molbev/msab279 PMC8763029 34542625

[82] Nayfach S, Roux S, Seshadri R, Udwary D, Varghese N, Schulz F, Wu D, Paez-Espino D, Chen IM, Huntemann M (2021). A genomic catalog of Earth's microbiomes. Nat Biotechnol.

[83] Shang J, Sun Y (2021). Predicting the hosts of prokaryotic viruses using GCN-based semi-supervised learning. BMC Biol.

[84] Ahlgren NA, Ren J, Lu YY, Fuhrman JA, Sun F (2017). Alignment-free $d_2;*$ oligonucleotide frequency dissimilarity measure improves prediction of hosts from metagenomically-derived viral sequences. Nucleic Acids Res.

[85] Edwards RA, McNair K, Faust K, Raes J, Dutilh BE (2016). Computational approaches to predict bacteriophage-host relationships. FEMS Microbiol Rev.

[86] Dzunkova M, Low SJ, Daly JN, Deng L, Rinke C, Hugenholtz P (2019). Defining the human gut host-phage network through single-cell viral tagging. Nat Microbiol.

[87] Koskella B, Meaden S (2013). Understanding bacteriophage specificity in natural microbial communities. Viruses.

[88] Pinnell LJ, Reyes AA, Wolfe CA, Weinroth MD, Metcalf JL, Delmore RJ, Belk KE, Morley PS, Engle TE (2022). **Bac**teroidetes and firmicutes drive differing microbial diversity and community composition among micro-environments in the bovine rumen. Front Vet Sci.

[89] Dao TK, Do TH, Le NG, Nguyen HD, Nguyen TQ, Le TT, Truong NH. Understanding the role of prevotella genus in the digestion of lignocellulose and other substrates in Vietnamese native goats' rumen by metagenomic deep sequencing. Animals (Basel). 2021;11(11):3257. 10.3390/ani11113257 PMC8614338 34827987

[90] Peng X, Wilken SE, Lankiewicz TS, Gilmore SP, Brown JL, Henske JK, Swift CL, Salamov A, Barry K, Grigoriev IV (2021). Genomic and functional analyses of fungal and bacterial consortia that enable lignocellulose breakdown in goat gut microbiomes. Nat Microbiol.

[91] Niwinska B: Digestion in Ruminants. In*.*, edn.; 2012: 245–258.

[92] Liu J, Taft DH, Maldonado-Gomez MX, Johnson D, Treiber ML, Lemay DG, DePeters EJ, Mills DA (2019). The fecal resistome of dairy cattle is associated with diet during nursing. Nat Commun.

[93] Gao NL, Zhang C, Zhang Z, Hu S, Lercher MJ, Zhao XM, Bork P, Liu Z, Chen WH (2018). MVP: a microbe-phage interaction database. Nucleic Acids Res.

[94] Chaumeil PA, Mussig AJ, Hugenholtz P, Parks DH (2019). GTDB-Tk: a toolkit to classify genomes with the Genome Taxonomy Database. Bioinformatics.

[95] Altermann E, Schofield LR, Ronimus RS, Beatty AK, Reilly K (2018). **Inh**ibition of rumen methanogens by a novel archaeal lytic enzyme displayed on tailored bionanoparticles. Front Microbiol.

