# Supplementary material for: A compendium of ruminant gastrointestinal phage genomes revealed a higher proportion of lytic phages than in any other environments
Source: Microbiome. 2024 Apr 4;12:69. doi: 10.1186/s40168-024-01784-2 (PMC10993611; doi:10.1186/s40168-024-01784-2)
Supplement: Supplementary file 3 — Additional file 2: Figure S1. Overview of the custom pipeline employed for the reconstruction of URPC. Figure S2. Phage quality assessment and annotation at different lengths. Figure S3. The number of phages identified along the ruminant gastrointestinal tract. Figure S4. The URPC shows longer length than viral genomes from human gut. Figure S5. Taxonomic annotation of the URPC genomes. Figure S6. Family annotation of RVD viral genomes by VirusTaxo and Demovir. Figure S7. Percentages of novel phages in URPC as compared with public datasets at 95% average nucleotide identity (ANI) thresholds. Figure S8. Lifestyle analysis of the phages identified in the ruminants of different GIT sites. [file 40168_2024_1784_MOESM2_ESM.pdf]

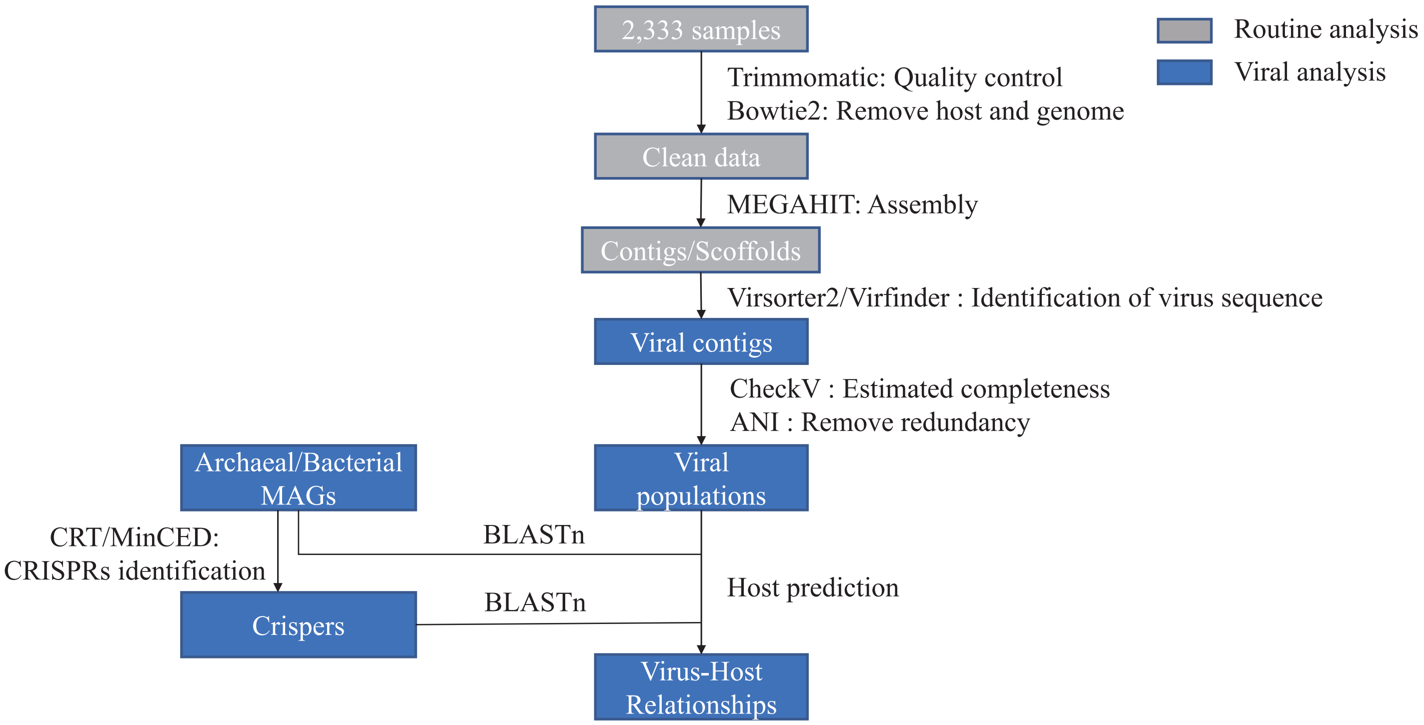

Figure S1.Overview of the custom pipeline employed for the reconstruction of URPC. Workflow of the phage-detection pipeline. Details of the pipeline are described in Methods

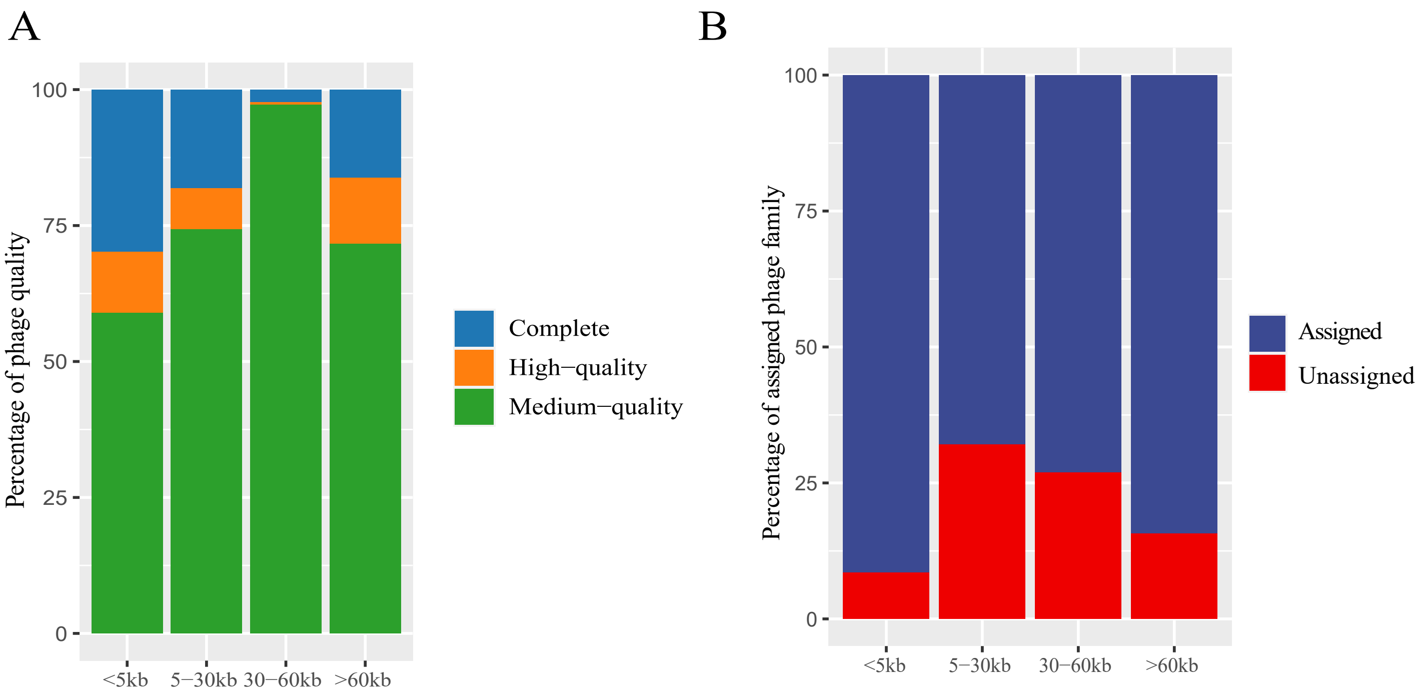

Figure. S2 Phage quality assessment and annotation at different lengths
A Percentages of phage quality (estimated by CheckV) at different lengths. **B** Percentage of assigned phage family at different lengths.

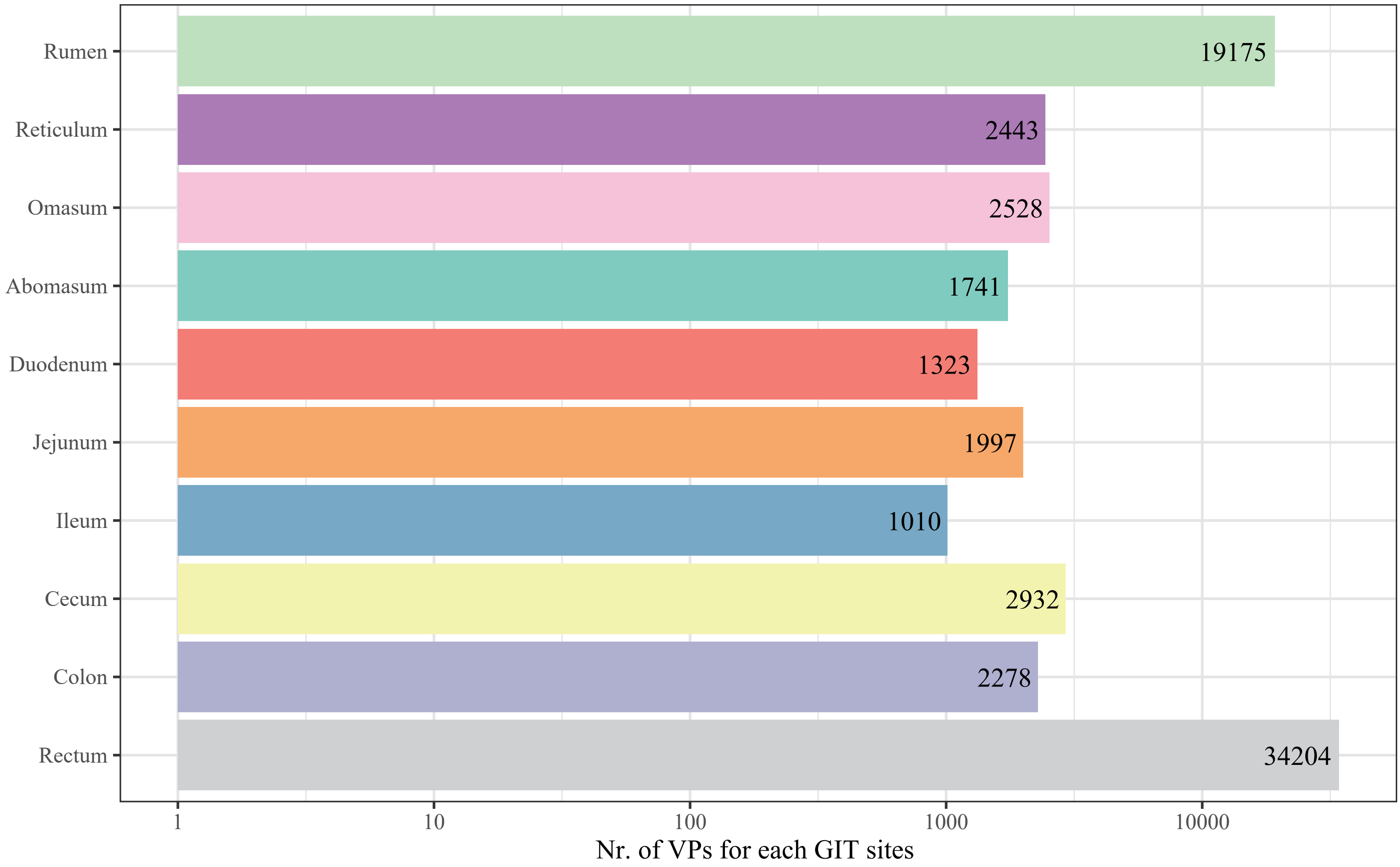

Figure S3. Histogram showing the number of phages identified along the ruminant gastrointestinal tract

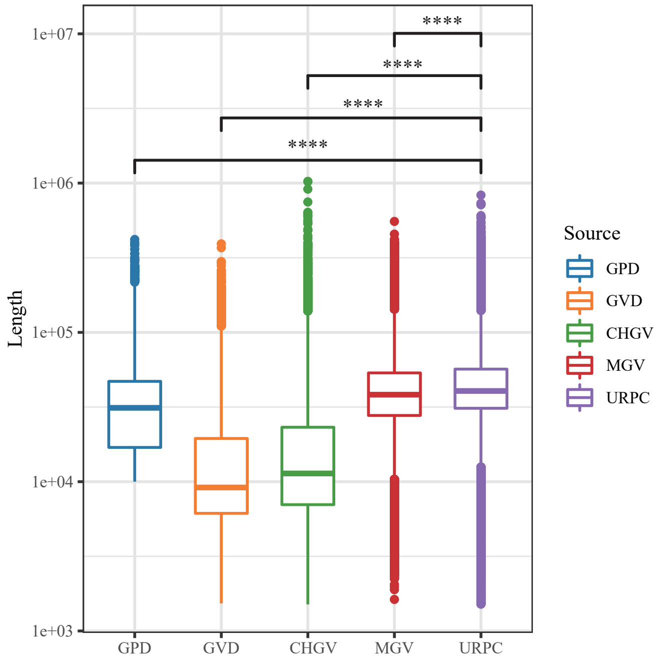

Figure S4. The URPC shows longer length than viral genomes from human gut. The boxplot showing the comparison of viral length between URPC and four viral databases from human gut. Wilcoxon Rank Sum test were used to show the statistical significance between groups. ns: no significance, * P < 0.05, ** P < 0.01, *** P < 0.0001, **** P < 0.0001

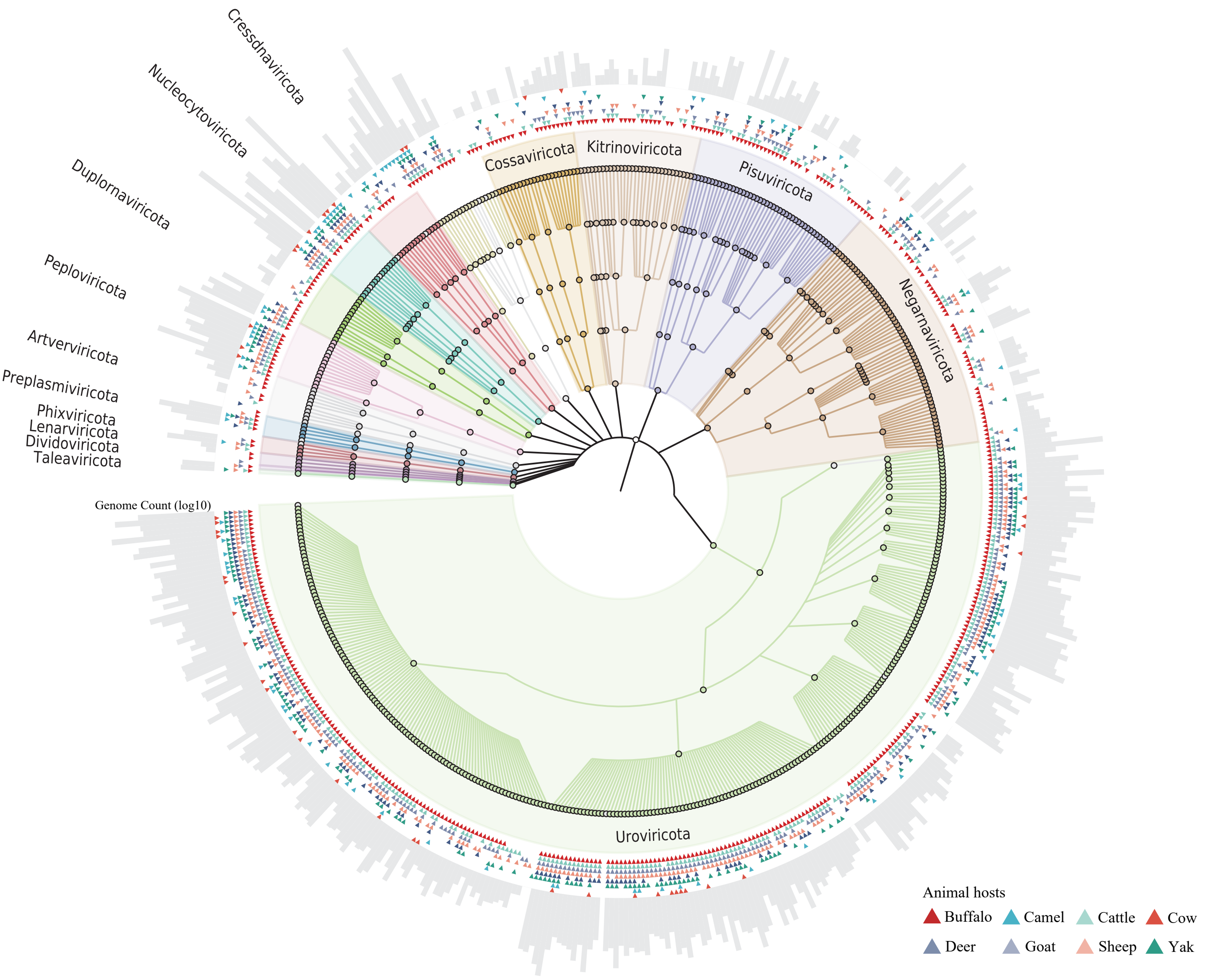

Figure S5. Taxonomic annotation of the URPC genomes. Phylogenetic relationship among the 64,922 phages classified rank by VirusTaxo and Demovir (see Methods), color-coded by the viral phyla. Histogram showing the number of phages were identified. The triangle symbol indicating the animal host of phages.

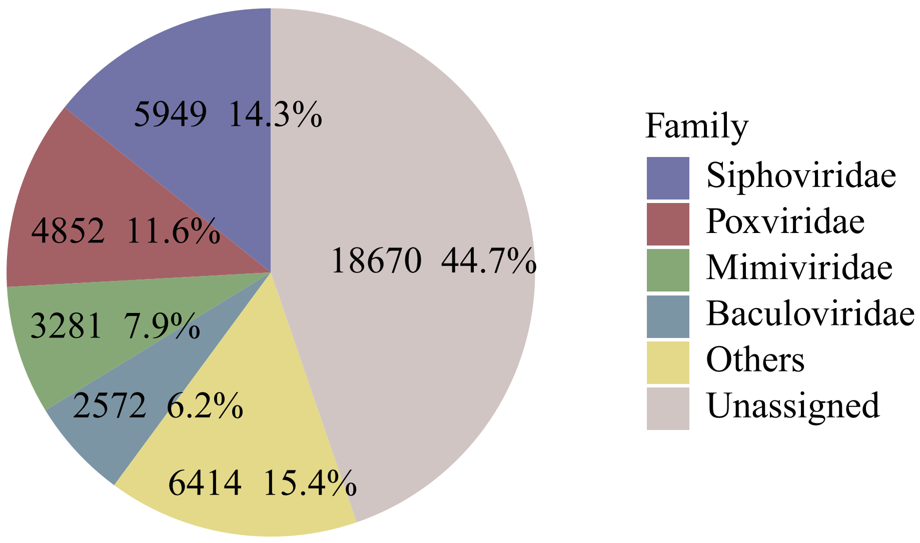

Figure. S6 Family annotation of RVD viral genomes by VirusTaxo and a Demovir script (Table S8, see Methods).

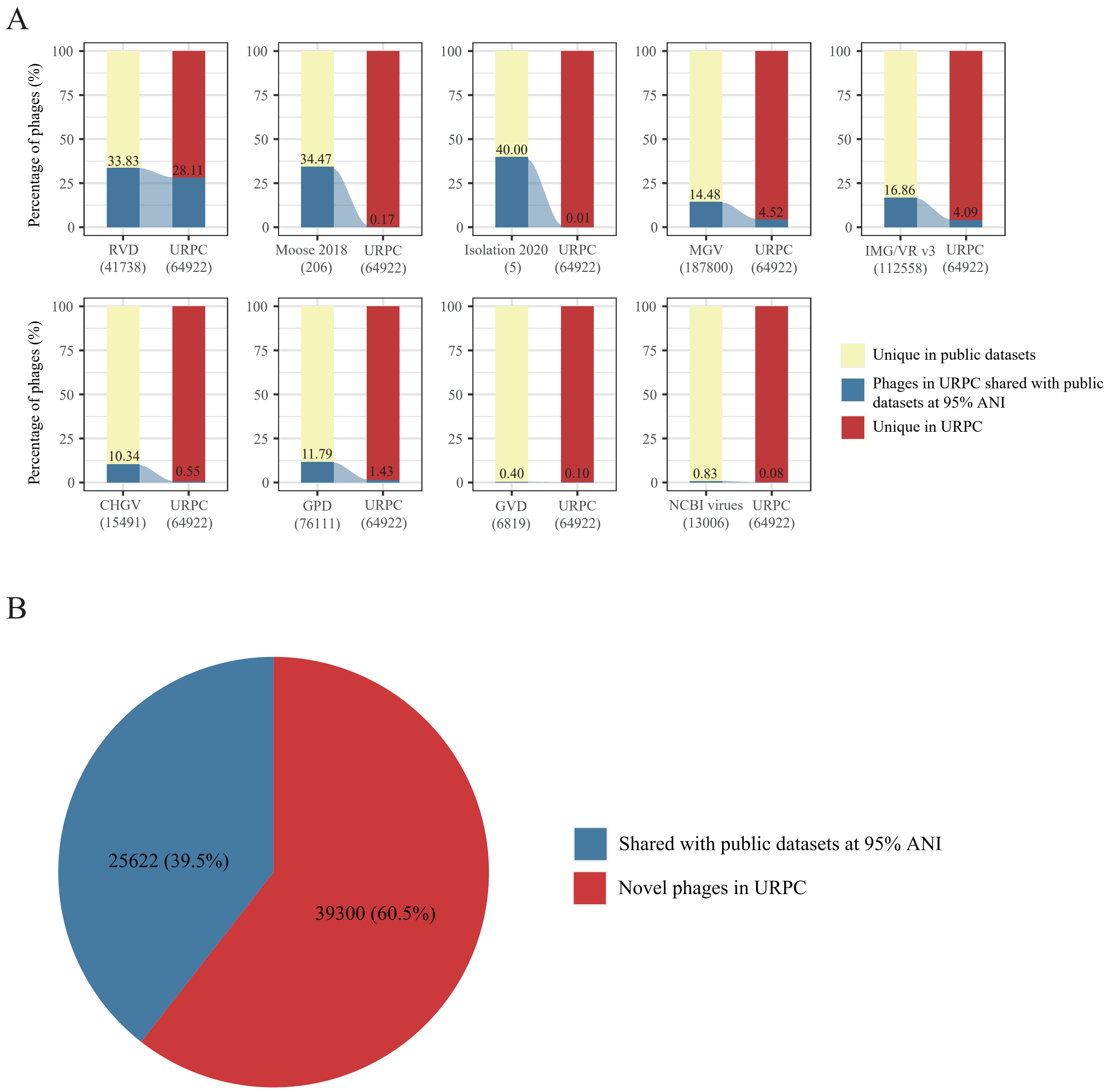

Figure. S7 Comparison of URPC similarity with public databases for viral genomes
A Percentages of shared and unique phages in URPC and other datasets that passed our filtering criteria (i.e., CheckV completeness score > 50% and length >1.5k) at a 95% average nucleotide identity (ANI) threshold. **B** Percentages of novel phages in URPC as compared with public datasets at 95% ANI threshold.

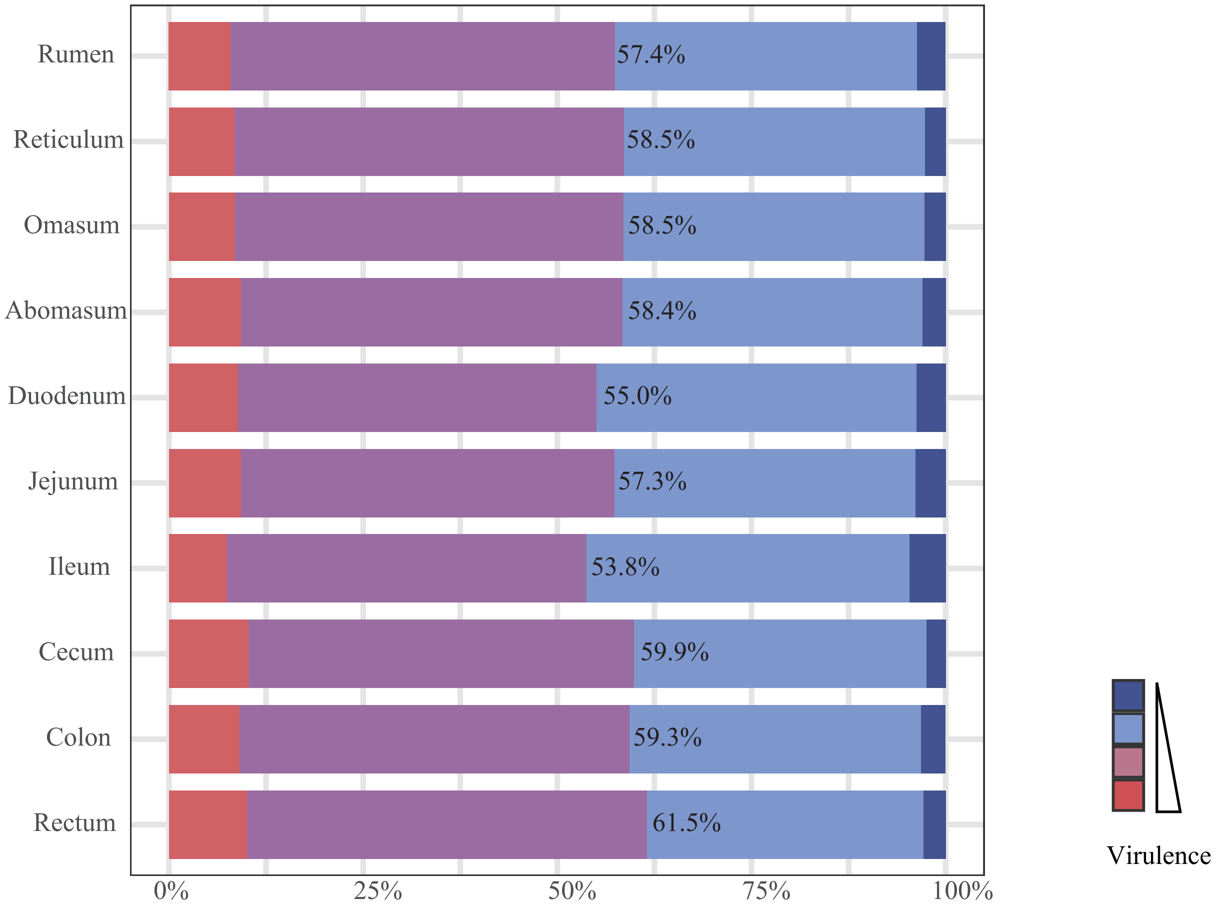

Figure. S8 Lifestyle analysis of the phages identified in the ruminants of different GIT sites.
